# Associations of Circulating Oxidized LDL and Conventional Biomarkers of Cardiovascular Disease in a Cross-Sectional Study of the Navajo Population

**DOI:** 10.1371/journal.pone.0143102

**Published:** 2016-03-03

**Authors:** Molly E. Harmon, Matthew J. Campen, Curtis Miller, Chris Shuey, Miranda Cajero, Selita Lucas, Bernadette Pacheco, Esther Erdei, Sandy Ramone, Teddy Nez, Johnnye Lewis

**Affiliations:** 1 Department of Pharmaceutical Sciences and Community Environmental Health Program, University of New Mexico, Albuquerque, New Mexico, United States of America; 2 Southwest Research and Information Center, Albuquerque, New Mexico, United States of America; Showa University School of Pharmacy, JAPAN

## Abstract

The prevalences of cardiovascular disease (CVD) and type 2 diabetes (T2D) have increased among the Navajo Native American community in recent decades. Oxidized low-density lipoprotein (oxLDL) is a novel CVD biomarker that has never been assessed in the Navajo population. We examined the relationship of oxLDL to conventional CVD and T2D risk factors and biomarkers in a cross-sectional population of Navajo participants. This cross-sectional study included 252 participants from 20 Navajo communities from the Diné Network for Environmental Health Project. Plasma samples were tested for oxLDL levels by a sandwich enzyme-linked immunosorbent assay. Univariate and multivariate analyses were used to determine the relationship of oxLDL and oxidized- to non-oxidized lipoprotein ratios to glycated hemoglobin (HbA1c), C-reactive protein (CRP), interleukin 6 (IL6) and demographic and health variables. Type 2 diabetes, hypertension and obesity are very prevalent in this Navajo population. HbA1c, CRP, body mass index (BMI), high-density lipoprotein, and triglycerides were at levels that may increase risk for CVD and T2D. Median oxLDL level was 47 (36.8–57) U/L. Correlational analysis showed that although oxLDL alone was not associated with HbA1c, oxLDL/HDL, oxLDL/LDL and CRP were significantly associated with HbA1c and glucose. OxLDL, oxLDL/HDL and oxLDL/LDL were significantly associated with CRP. Multivariate analysis showed that triglycerides were a common and strong predictor of oxLDL, oxLDL/HDL and oxLDL/LDL. OxLDL was trended with HbA1c and glucose but did not reach significance, however, HbA1c was an independent predictor of OxLDL/HDL. CRP trended with oxLDL/HDL and was a weak predictor of oxLDL/LDL. This Navajo subset appears to have oxLDL levels comparable to subjects without evidence of CVD reported in other studies. The high prevalence of T2D, hypertension and obesity along with abnormal levels of other biomarkers including HbA1c indicate that the Navajo population has a worsening CVD risk profile.

## Introduction

Cardiovascular diseases (CVD) and type 2 diabetes (T2D) were rarely reported in the Navajo population until the 1930s [1]. In recent decades, the prevalences of cardiovascular related health conditions such as T2D, overweight and hypertension have increased [2–6], with CVD being the leading cause of non-accidental death among Navajos [3,6], as well as in other Native American populations [7,8]. It has been well-established that diabetes increases the risk for CVD, and Navajo are now developing T2D at a rate four times higher than the United States average [5]. In 1997, the last published comprehensive look at Navajo-specific health status, the Navajo Health and Nutrition Survey, reported that nearly 40% of Navajos over the age of 45 had T2D [5]. According to a recent Center for Disease Control report, the rate of new T2D cases among American Indian/Alaskan native youth aged 10–19 is higher than any other ethnic group or race in the United States (U.S.) [2]. T2D, along with obesity and hypertension, have become major public health concerns in a population that has become increasingly at risk for CVD.

Clinically, numerous circulating biomarkers including C-reactive protein (CRP) and interleukin-6 (IL6) have been useful in predicting CVD outcomes and assessing risk [9,10]. CRP has long been established as a marker of chronic systemic inflammation and is regulated by IL6, a pro-inflammatory cytokine and important inducer and regulator of chronic inflammation [11,12]. Novel biomarkers for CVD are emerging including oxidized LDL (oxLDL) cholesterol. OxLDL is increased in subclinical atherosclerosis [13] and is often a stronger predictor of acute coronary artery disease (CAD) than standard lipid measures or other conventional risk factors [14]. OxLDL levels are reportedly able to distinguish patients with CAD from healthy cohorts [15], and serve as a predictor of future myocardial infarction in patients with unstable CAD [16]. Oxidized LDL is also associated with T2D [17]. More research is needed to establish oxLDL as a clinically useful biomarker.

The relationship between oxLDL and CVD risk factors and biomarkers in the Navajo community is unknown. Given the high prevalence of T2D in this population, our purpose was to characterize CVD biomarkers in a cross-sectional Navajo population and to evaluate the association of oxLDL with HbA1C, widely accepted as a major biomarker of glycemic control, ultimately to better understand how these metrics currently trend in an understudied ethnic group.

## Methods

### Study Population and Survey Methods

The geographic study area for the study population was located in the northwestern region of New Mexico, in the political division of the Navajo Nation known as the Eastern Agency. Demographic and health data were obtained from the Water and Land Use, Environmental and Health Survey, designed by the Diné Network for Environmental Health (DiNEH) Project [18,19] which implemented a community based participatory approach to enroll 1,304 participants from 20 Chapters of the Navajo Nation. The DiNEH Project was originally intended to evaluate kidney disease in the Navajo due to potential exposures to uranium from numerous abandoned uranium mines, but the study was later expanded to evaluate the overall health of this population, including cardiovascular, immunological and neoplastic diseases. Surveys were administered by the DiNEH Project in interview fashion to participants between 2005 and 2010. Participants reported CVD related health conditions on the surveys. Height and weight were measured at the time of survey administration to determine body mass index (BMI). From these DiNEH participants, we had a recruitment goal for a follow-up blood collection of 450 participants; we had a 40–90% recruitment success rate on any given day from 2010–2011 for a final total of 252 Navajo participants. Non-fasting plasma samples were collected along with clinical assessments conducted by a Navajo Area Indian Health Service mobile unit at multiple community locations. All serum biomarker data were derived from this subset. Blood pressure was measured by automatic cuff. All participants provided written informed consent, and the study was approved by both the University of New Mexico Human Research Review Committee and the Navajo Nation Human Research Review Board.

### Biomarker Measurements

Plasma samples were tested for oxLDL by a sandwich enzyme-linked immunosorbent assay, according to manufacturer’s instructions (Mercodia, Uppsala, Sweden) (n = 252). The full volume of intended blood samples was not available for all participants so subsequent analyses were prioritized resulting in the N noted below for each of the markers reported herein. Interleukin 6 (IL6) was determined by MILLIPLEX ultrasensitive human magnetic bead set (Millipore Inc; Billerica, MA) and multiplexing technology to obtain serum cytokine concentration measures (in pg/ml) (n = 236). Magnetic bead detection was carried out on a MAGPIX machine and multiplexing platform capable of performing quantitative analysis of low concentration of protein markers (Luminex Corporation, Austin, TX). Analytical values were determined by xPONENT 4.2 Software (Luminex Corporation, Austin, TX) by standards provided in the assay kit for each cytokine. All remaining biochemical analyses were performed by a reference laboratory (LabCorp, Phoenix, AZ), reported to Indian Health Services and compiled in a clinical database (RedCAP). Glycated hemoglobin (HbA1c) (n = 249) was determined by the Roche Tina-quant assay. CRP (n = 249) was assessed quantitatively by latex immunoturbidimetry. There were 217 samples available for assessing total serum cholesterol, high-density lipoprotein (HDL), and triglycerides (TG), which were measured by conventional clinical analytical methods; LDL was measured directly (n = 211).

### Statistical Analysis

Descriptive summary statistics were reported as median (interquartile range; IQR) for continuous variables, unless otherwise indicated. Non-Gaussian distributions were normalized using a logarithmic transformation. Pearson correlations and multivariate analysis were performed to assess the relationship of oxLDL to other CVD and T2D risk factors and biomarkers. A value of P < 0.05 was considered statistically significant. Statistical analyses were performed using R version 2.12.1 (The R Foundation for Statistical Computing, 2010, 64-bit) and GraphPad Prism 6.0 (GraphPad Software, La Jolla, CA, USA). All multivariate models were adjusted for age, gender, BMI, total cholesterol, triglycerides, diastolic and systolic blood pressures, non-fasting glucose, HbA1c, CRP, and IL6. The model for oxLDL/HDL was also adjusted for LDL cholesterol, and the oxLDL/LDL model was adjusted for HDL cholesterol. Model selection was performed based on AIC (Akaike Information Criterion) or BIC (Bayesian Information Criterion).

## Results

### Clinical Characteristics and Prevalence of Health Conditions in the Navajo Population

Table 1 shows the characteristics of the Navajo population subset (n = 252) relative to the original DiNEH study mean age of 55.3 ±14.3 years versus 51.5 ±17.4 years, respectively; both were similar in gender proportion, BMI and self-reported health conditions. Diabetes and hypertension were the predominant conditions self-reported by both the subset and original DiNEH population, at 26.2% (subset) and 25.1% (DiNEH) for diabetes and 38.1% (subset) and 35.9% (DiNEH) for hypertension; heart disease, stroke and myocardial infarction were less prevalent in both groups.

**Table 1:** Characteristics and clinical parameters of the Navajo subset and original DiNEH participants.

	DiNEH Subset		DiNEH Participants*
Variable		n	(n = 1304)
Age, years	55.3 ± 14.3	252	51.5 ± 17.4
Female, %	57.5	252	56.4
Self-reported health conditions		252	
Type 2 Diabetes, %	26.2		25.1
Hypertension, %	38.1		35.9
Heart Disease, %	6		5.4
Myocardial Infarction, %	4.4		3.1
Stroke, %	5.2		3.5
Body mass index (BMI), kg/m^2^	29.7 (26.8–33.6)	252	28.3 (25.1–32.6)
Underweight (BMI ≤ 18.4), %	0.8		0.7
Normal (BMI = 18.5–24.9), %	14.5		23.0
Overweight (BMI = 25.0–29.9), %	37.1		35.0
Obese (BMI ≥ 30), %	47.6		41.2
Total Cholesterol, mg/dL	182 (162–204)	217	
LDL, mg/dL	105 (90–123)	211	
HDL, mg/dL	45 (38–54)	217	
Triglycerides, mg/dL	183 (127–251)	217	
Systolic BP (mmHg)	129.5 (117–143)	248	
Diastolic BP (mmHg)	78 (71–86)	248	
CRP, mg/L	2.1 (0.9–4.8)	249	
CRP > 3.0 mg/L, %	38.5		
oxLDL, U/L	46.9 (36.8–57)	252	
IL6, pg/ml	5.9 (1.8–12.5)	236	
Glucose (non-fasting), mg/dL	91 (78–121)	249	
HbA1c, %	6.2 (5.8–7.1)	249	
% Normal (≤ 5.6%)	15.7		
% Pre-diabetes (5.7–6.4%)	45.8		
% Diabetes (≥ 6.5%)	38.6		

*Biomarker levels and blood pressure were not available for the original DiNEH participants. Data are presented as median (IQR) or %. LDL, low-density lipoprotein; HDL, high-density lipoprotein; BP, blood pressure; HbA1c, glycated hemoglobin; CRP, C-reactive protein; oxLDL, oxidized low-density lipoprotein; IL6, interleukin 6.

The prevalence of obesity (BMI ≥30 kg/m^2^) in the Navajo subset was 47.6%, which was higher than the prevalence in the original DiNEH study population (41.2%) (Table 1). Over one-third of both study populations were overweight (BMI 25.0–29.9 kg/m^2^). Furthermore, the majority of participants in both the subset (84.7%) and original DiNEH population (76.2%) had BMI values considered either “overweight” or “obese.” Only 23% of original DiNEH participants and 14.5% of the subset were within a normal BMI range (18.5–24.9 kg/m^2^).

Median systolic blood pressure in the Navajo subset was pre-hypertensive; for 68% of this study population, systolic pressures were at or above pre-hypertensive levels (>120 mmHg) according to American Heart Association guidelines [20] (Table 1). Median diastolic blood pressure was normal although almost half (47%) of the subset had diastolic pressures that were measured at or above pre-hypertensive levels (>80 mmHg). Blood pressure measurements were not available for the original DiNEH population. Thirty-five percent of the subset reported taking hypertension medication.

### Levels of Biomarkers Associated with CVD and T2D

Plasma biomarker levels for the Navajo subset are shown in Table 1. The median oxLDL level in the Navajo subset was 46.9 (36.8–57) U/L. The median IL6 level was 5.9 (1.8–12.5) pg/ml. Currently, reference ranges for oxLDL and IL6 as biomarkers have not been established. The median CRP level was 2.1 (0.9–4.8) (average risk), however, 38.5% had levels greater than 3.0 mg/L, which is considered high risk for heart disease [21]. Circulating lipid levels are shown in Table 1. Based on American Heart Association guidelines [22] median total cholesterol in this subset was “desirable,” and LDL cholesterol levels were near optimal. However, HDL cholesterol was bordering on low, and triglycerides were high; both are risk factors for heart disease. No data were available for the use of lipid-lowering medications in this subset.

**Fig 1:**
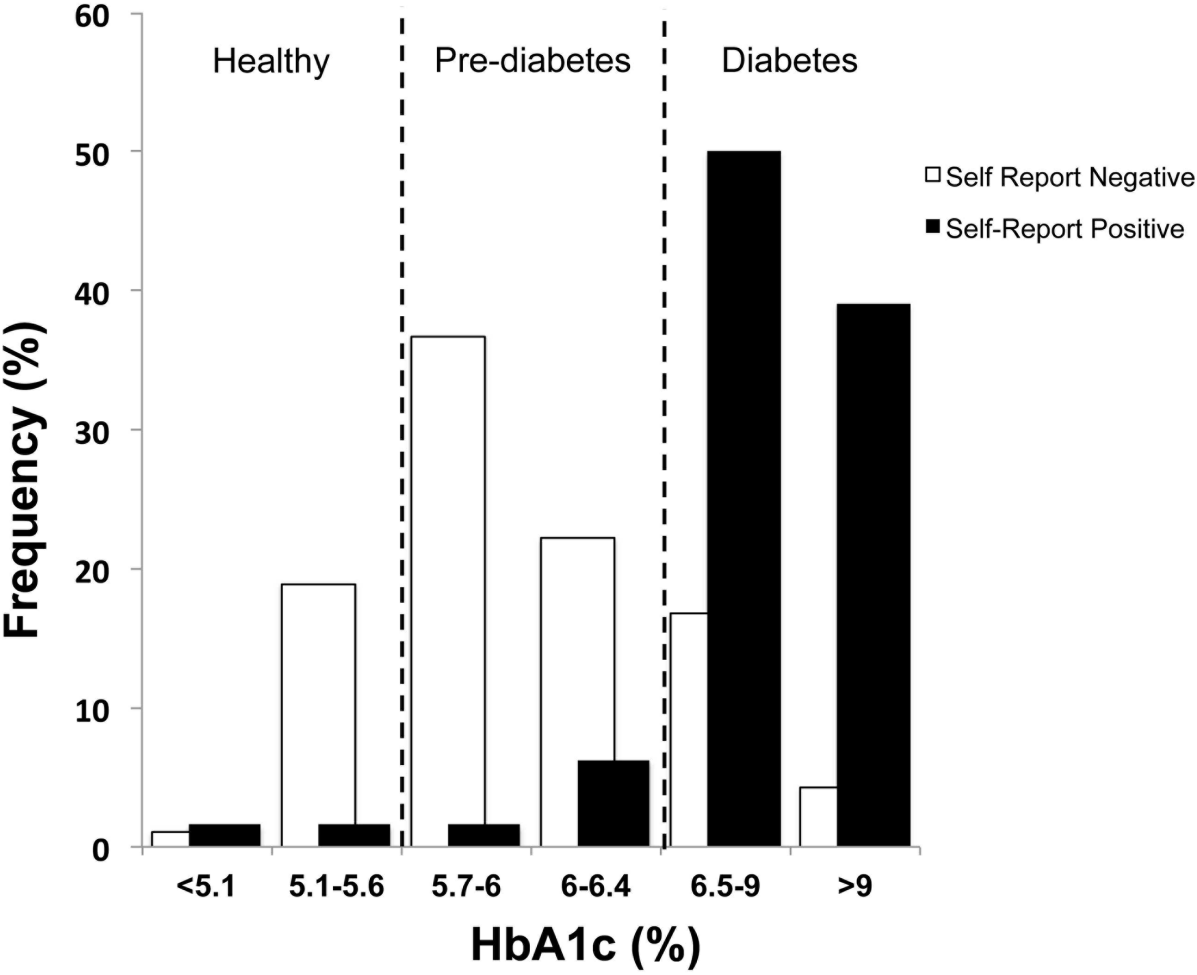
Frequency histogram showing survey participants self-reporting a diagnosis of either type 2 diabetes (positive) or no type 2 diabetes (negative) plotted against participants’ actual HbA1c levels. Dashed lines correspond to HbA1c category cutoffs. Normal: HbA1c ≤ 5.6%; Pre-diabetes: HbA1c = 5.7–6.4%; Diabetes ≥ 6.5%.

As mentioned, a diagnosis of T2D was self-reported by 26.2% of the Navajo subset (Table 1), however, our data indicate that 84% of HbA1c levels in the Navajo subset as a whole were pre-diabetic (5.7–6.4%) or higher (Table 1, Fig 1), as classified by the American Diabetes Association [23]. Indeed, the median HbA1c in the Navajo subset was pre-diabetic at 6.2% (5.8–7.1%). Furthermore, as shown in Fig 1, of those who denied a diagnosis of diabetes at the time of survey administration, 59% had pre-diabetic levels of HbA1c with a mean of 6.3% ± 1.3. HbA1c levels greater than 6.5% are indicative of diabetes. For participants self-reporting a diagnosis of diabetes, 89% were in the diabetic range with a mean HbA1c of 8.9% ± 2.5. As a whole, more than a third (39%) of the subset had diabetic levels of HbA1c, and nearly half (46%) were in the pre-diabetic range (Table 1). Only 16% were in the normal HbA1c range (<5.6%). The median non-fasting glucose of this Navajo subset was 91 (78–121) mg/dl (Table 1), which is comparable to a normal fasting glucose level. Twenty-five percent of the subset reported taking oral medication and/or insulin for diabetes.

### Oxidized LDL Interactions

Table 2 shows the Pearson correlations of oxLDL and oxidized- to non-oxidized lipoprotein ratios (oxLDL/HDL and oxLDL/LDL) to more conventional risk factors and biomarkers of CVD and T2D in this Navajo subset of volunteers. OxLDL trended positively with HbA1c but did not reach significance. OxLDL was moderately correlated with non-fasting glucose but negatively correlated with self-report of diabetes. When oxLDL levels were compared between normal, pre-diabetic, and diabetic levels of HbA1c, no differences were found; the same was true for the oxidized-to non-oxidized lipoprotein ratios (data not shown). Both oxLDL/HDL and oxLDL/LDL were moderately correlated with HbA1c and non-fasting glucose. CRP, but not IL6, correlated moderately with HbA1c and non-fasting glucose. HbA1c was strongly correlated with non-fasting glucose and self-report of diabetes. OxLDL and oxLDL/HDL were strongly correlated with DBP, and CRP was weakly correlated with DBP, but HbA1c was the only biomarker associated with a self-report of high blood pressure. OxLDL/LDL was significantly correlated with self-report of heart attack. OxLDL, oxLDL/HDL, and oxLDL/LDL correlated significantly with each other and with all conventional lipid biomarkers, total cholesterol (TC), triglycerides (TG), HDL and LDL (with the exception that oxLDL/LDL did not correlate with TC). HbA1c was weakly correlated with TG and negatively correlated with HDL. CRP was also negatively correlated with HDL. IL6 was weakly correlated with TC and negatively correlated with TG. OxLDL, oxLDL/HDL and oxLDL/LDL were moderately correlated with CRP. OxLDL was negatively correlated with IL6. CRP was moderately correlated with IL6. OxLDL/HDL and CRP were negatively correlated with age. HbA1c and CRP were moderately correlated BMI. HbA1c was the only biomarker correlated with gender.

**Table 2:** Univariate relationships between novel CVD biomarkers (oxLDL, oxLDL/HDL, oxLDL/LDL) with conventional biomarkers (CRP, IL6, HbA1c), risk factors of CVD and T2D and self-reported health conditions.

Variable	oxLDL		oxLDL/HDL		oxLDL/LDL		CRP		IL6		HbA1C	
	r	p	r	p	r	p	r	p	r	p	r	p
Age (years)	-0.070	0.268	**-0.135**	**0.046**	-0.065	0.350	**-0.208**	**0.001**	0.018	0.773	0.104	0.100
Gender	0.020	0.756	-0.111	0.104	-0.001	0.986	0.064	0.315	-0.005	0.934	**0.146**	**0.021**
BMI (kg/m^2^)	0.004	0.947	0.058	0.392	0.036	0.602	**0.235**	**<0.0001**	0.115	0.067	**0.157**	**0.013**
Hypertension*	-0.030	0.638	0.034	0.616	0.109	0.116	-0.026	0.679	0.064	0.314	**0.294**	**<0.0001**
Heart attack*	-0.014	0.822	0.026	0.701	**0.141**	**0.040**	-0.006	0.920	0.029	0.645	0.086	0.177
Diabetes*	**-0.127**	**0.044**	-0.035	0.603	0.060	0.385	0.013	0.836	0.096	0.130	**0.596**	**<0.0001**
TC (mg/dl)	**-0.409**	**<0.0001**	**-0.744**	**<0.0001**	-0.434	0.492	-0.186	0.666	**0.056**	**0.032**	-0.143	0.679
TG (mg/dl)	**0.592**	**<0.0001**	**0.363**	**<0.0001**	**-0.377**	**<0.0001**	0.005	0.274	**-0.125**	**0.024**	**0.031**	**0.019**
HDL (mg/dl)	**0.095**	**<0.0001**	**0.118**	**<0.0001**	**0.006**	**<0.0001**	**-0.008**	**0.006**	-0.003	0.412	**-0.003**	**0.037**
LDL (mg/dl)	**0.165**	**<0.0001**	**0.209**	**<0.0001**	**0.067**	**<0.0001**	0.160	0.943	-0.028	0.071	0.023	0.652
SBP (mmHg)	0.614	0.137	0.389	0.085	-0.048	0.929	0.030	0.904	-0.145	0.964	0.028	0.963
DBP (mmHg)	**0.564**	**0.009**	**0.700**	**0.002**	0.457	0.340	**0.075**	**0.012**	-0.153	0.658	0.160	0.715
log oxLDL (U/L)	…	…	**0.829**	**<0.0001**	**0.523**	**<0.0001**	…	…	…	…	…	…
log CRP (mg/L)	**0.147**	**0.021**	**0.167**	**0.014**	**0.147**	**0.034**	…	…	…	…	…	…
log IL6 (pg/ml)	**-0.163**	**0.009**	-0.121	0.076	-0.076	0.272	**0.249**	**<0.0001**	…	…	…	…
log HbA1C (%)	0.118	0.062	**0.172**	**0.012**	**0.138**	**0.047**	**0.196**	**0.002**	0.084	0.189	…	…
Glucose (mg/dl)	**0.163**	**0.010**	**0.203**	**0.003**	**0.186**	**0.007**	**0.209**	**0.001**	0.027	0.667	**0.826**	**<0.0001**

*Participant self-reported health conditions; heart disease and stroke did not correlate with any biomarkers. Pearson correlation coefficients (r). P < 0.05 was considered statistically significant (bold). Non-Gaussian distributions were log transformed. CVD, cardiovascular disease; oxLDL, oxidized low-density lipoprotein; LDL, low-density lipoprotein; HDL, high-density lipoprotein; HbA1c, glycated hemoglobin; CRP, C-reactive protein; IL6, interleukin 6, SBP, systolic blood pressure; DBP, diastolic blood pressure; BMI, body mass index

### Multivariate analysis

Multivariate analysis confirmed that oxLDL was independently and significantly associated with total cholesterol, LDL and triglycerides but not CRP or IL6. OxLDL was marginally associated with age, HbA1c and glucose, but these associations did not reach significance (Table 3).

**Table 3:** Coefficient estimates of final models for oxLDL, oxLDL/HDL and oxLDL/LDL.

	OxLDL			OxLDL/HDL			OxLDL/LDL		
Variable	Estimate	Error	*P*	Estimate	Error	*P*	Estimate	Error	*P*
Age	-0.112	0.061	0.067	-0.005	0.002	0.009			
Gender	-0.710	1.590	0.656	0.107	0.052	0.041			
BMI	-0.230	0.147	0.120				-0.003	0.001	0.046
SBP	0.052	0.051	0.303						
DBP	-0.061	0.082	0.456						
TC	0.168	0.064	0.010	-0.006	0.001	<0.0001	-0.001	0.0002	<0.0001
LDL	0.140	0.057	0.015	0.010	0.001	<0.0001			
HDL	-0.317	0.086	0.0003						
TG*	6.254	2.963	0.036	0.851	0.059	<0.0001	0.169	0.016	<0.0001
HbA1c	0.636	0.361	0.080	0.062	0.023	0.0094			
Glucose*†	2.750	1.437	0.057	-0.203	0.098	0.039			
CRP*	0.454	0.669	0.498	0.042	0.022	0.057	0.014	0.006	0.028
IL6*	-1.013	0.809	0.212						
n	191			204			206		
Adjusted R^2^	0.649			0.638			0.351		
AIC	1426.084			161.012			-336.174		
BIC	1471.616			194.193			-316.206		

P < 0.05 was considered statistically significant.

*Non-Gaussian distributions were log transformed.

^†^Non-fasting. OxLDL, oxidized low-density lipoprotein; LDL, low-density lipoprotein; HDL, high-density lipoprotein; BMI, body mass index; SBP, systolic blood pressure; DBP, diastolic blood pressure; TC, total cholesterol; TG, triglycerides; HbA1c, glycated hemoglobin; CRP, C-reactive protein; IL6, interleukin 6

Triglycerides were a very strong predictor of oxLDL/HDL. OxLDL/HDL was independently and positively associated with HbA1c but negatively associated with glucose. OxLDL/HDL was weakly and negatively associated with age and total cholesterol and positively associated with LDL. OxLDL/HDL trended positively with CRP but did not reach significance. OxLDL/LDL was independently associated with triglycerides and weakly associated with CRP. OxLDL/LDL was negatively associated with total cholesterol and BMI (Table 3).

## Discussion

Our goal was to measure oxLDL and examine its relationship to health characteristics and T2D in a cross-sectional subset of the Navajo Nation, as oxLDL has never been assessed in this population. The median oxLDL level in this subset was 47 U/L (IQR: 36.8–57) and ranged from 12.7–138.2 U/L. The mean oxLDL level (48.5 ± 17.0 U/L) was similar to mean oxLDL levels measured in subjects without evidence of atherosclerosis reported in other studies [S1 Table; 14, 24–27] that also used the same murine monoclonal antibody 4E6 based ELISA [28]. However, oxLDL levels varied widely in these studies and consistent associations related to CAD or diabetes disease status are difficult to discern.

Increased levels of HbA1c, a biomarker for T2D, have been associated with CAD, overall CVD, and all-cause mortality [29,30]. For the present Navajo subset, the median HbA1c level was in the pre-diabetic range with the majority of participants having pre-diabetic (46%) or diabetic (39%) levels of HbA1c. Other studies have shown an association between oxLDL and HbA1c [17], but in the present study univariate analysis showed that the oxLDL/HDL and oxLDL/LDL ratios correlated significantly with HbA1c, while oxLDL itself correlated only weakly with HbA1c (p = 0.062). We examined the ratios of oxLDL/HDL and oxLDL/LDL as previous reports suggest that these metrics may be more informative than oxLDL alone [31–33]. In a similar study of T2DM, oxLDL/HDL and oxLDL/LDL, but not oxLDL alone, were associated with atherosclerosis, even when adjusting for BMI, gender, age and hypolipidemic treatment [27]. LDL derived from diabetics is more susceptible to oxidation, and this susceptibility is associated with HbA1c levels [34,35]. The present Navajo subset also showed a similar lipid profile as seen in metabolic syndrome/diabetes, wherein LDL is normal, but HDL is low and triglycerides are high [4,36,37]. Thus, although LDL may be lower, it may also be more susceptible to oxidation further increasing CVD risk in this population.

T2D (26%) and hypertension (38%) in the Navajo subset and original DiNEH population (25% and 36%, respectively) have increased compared to previous reports [4,5] and are notably greater than U.S. prevalences [2,38]. Overweight and obesity continue to be very prevalent (at least 76%) in the Navajo population. In the Navajo subset, BMI was moderately correlated with CRP and HbA1c but not oxLDL or oxidized- to non-oxidized lipoprotein ratios. OxLDL was correlated with both CRP and IL6, suggesting a potential role for the contribution of overall inflammation to the health status of this population. CRP was also correlated with oxLDL/HDL and oxLDL/LDL. The Navajo subset exhibited moderate CRP levels (2.1 mg/dl; IQR: 0.9–4.8), but over 38% had levels greater than 3.0 mg/dl consistent with increasing risk for CVD. Mechanistic studies have demonstrated that CRP and oxLDL may be interacting in concert to promote atherosclerosis [39,40]. Despite low self-report of heart disease, heart attack and stroke, these data suggest that CVD- and T2D-related conditions may become more prevalent in this middle-aged Navajo population.

It is not surprising that standard lipid measures are predictive overall of oxLDL and oxLDL ratios with HDL and LDL. Triglycerides in particular were positively associated with all three response-variables, which is consistent with other studies that have assessed lipid oxidation in diabetes [41–43]. HbA1c seems to be somewhat predictive of oxLDL/HDL and perhaps oxLDL but not oxLDL/LDL. Non-fasting glucose was negatively associated with oxLDL/HDL but slightly positively associated with oxLDL possibly reflecting more of a post-prandial response. It has been suggested that LDL oxidation is associated with dyslipidemia as diabetes progresses [44]. Although not measured in this study, small dense LDL particles, HDL abnormalities and diminished serum anti-oxidative capacity in diabetics are strongly associated with metabolic syndrome and diabetes [41–43,45]. Inflammation is a component of both atherogenesis and the development of diabetes. CRP was somewhat predictive of the oxLDL ratios with HDL and LDL but not of oxLDL itself. Age was negatively associated with oxLDL and oxLDL/HDL suggesting that these variables decrease in middle age in the Navajo population. OxLDL/HDL appears to be higher in women.

### Study Limitations

An important limitation of our study was that some plasma obtained for biomarker assessments (2010–2011) was collected at the end of the survey administration (2005–2010). Thus, subjects may not have initially reported health conditions for which they were later diagnosed. Although review of medical records was attempted, complete records were not available for all volunteers so not all self-reported health conditions could be validated. Navajo also engage in traditional medical practices, which would not be included in clinical medical records. However, for the self-reported measures of BMI (data not shown) and diabetes where sufficient clinical data were available at the time of blood collection, concordance with self-report was >80% with errors. Thus, given that the time between survey and clinical assessment could range as long as 5 years, observed discrepancies were consistent with a clinical finding of disease subsequent to the survey. Although bias may present due to self-selection, the Navajo subset was similar to the larger DiNEH study population based on age, gender, BMI and self-reported disease conditions. Non-fasting blood samples may impact some of the biomarker levels, however, non-fasting lipid levels may better predict risk of cardiovascular events [46]. Participant use of diabetic and hypertensive medications was not included in our initial analysis.

The cross-sectional nature of this study does not allow for the determination of causality. The results of this study cannot be generalized to other populations. Finally, this population is known to have significant environmental exposure risks to metals from the legacy of uranium mining. However, the study population reported herein spans the full range of those exposures, including a significant proportion of unexposed participants [47], improving the generalizability of our results.

### Conclusions

The Navajo population faces a dramatic increase in overall CVD risk profile. This population is greatly in need of better control of diabetes, hypertension and obesity to prevent future CVD-related health complications. Concentrations of the CVD marker oxLDL in the Navajo population trend with indices of metabolic syndrome and T2D, but were consistent with several other published cohorts. The relationship between oxLDL and absolute CVD risk in individuals with pre-diabetes and diabetes from this underserved ethnic group remains unclear. Ongoing and future analyses are exploring the potential role of chronic environmental exposures and other possible socioeconomic and lifestyle risk factors in contributing to cardiometabolic disease.

## Supporting Information

S1 Table Comparison of Navajo subset mean oxLDL levels, age, BMI, and HbA1c and/or diabetes status with other studies using monoclonal antibody 4E6 to measure circulating oxLDL. (DOCX)

## Abbreviations

CAD: Coronary artery disease
CRP: C-reactive protein
CVD: Cardiovascular disease
DiNEH: Diné Network for Environmental Health
HBA1C: Hemoglobin A1c
HDL: High density lipoprotein
IL6: Interleukin-6
LDL: Low density lipoprotein
OxLDL: Oxidized low density lipoprotein
T2D: Type 2 diabetes
TC: Total cholesterol
TG: Triglycerides
U.S.: United States of America
