# Supplementary material for: Associations of Circulating Oxidized LDL and Conventional Biomarkers of Cardiovascular Disease in a Cross-Sectional Study of the Navajo Population
Source: PLoS One. 2016 Mar 3;11(3):e0143102. doi: 10.1371/journal.pone.0143102 (PMC4777541; doi:10.1371/journal.pone.0143102)
Supplement: S1 Table — (DOCX) [file pone.0143102.s001.docx]

**Supplemental Table 1** Comparison of Navajo subset mean oxLDL levels, age, BMI, and HbA1c and/or diabetes status with other studies using monoclonal antibody 4E6 to measure circulating oxLDL

| **Population studied** | **oxLDL (U/L)** | **Age (yrs)** | **BMI (kg/m^2^)** | **HbA1c (%)** | **Diabetes (%)** | **Participants (n)** | **Country** | **Ref.** |
| --- | --- | --- | --- | --- | --- | --- | --- | --- |
| *Navajo population* | *48.5* | *55.0* | *30.0* | *7.0* | *38.6* | *252* | *U.S.* |  |
| General population |  |  |  |  |  |  | Spain | 24* |
| No CAD event | 53.2 | 49.5 | 27.0 | n/a | 11.4 | 2690 |  |  |
| CAD event | 65.1 | 60.0 | 29.1 | n/a | 26.0 | 103 |  |  |
| No subclinical atherosclerosis | 51.8 | 45.4 | 26.9 | n/a | 7.9 | 1121 |  |  |
| Subclinical atherosclerosis | 57.8 | 58.0 | 28.3 | n/a | 16.7 | 306 |  |  |
| Patients w/o CVD |  |  |  |  |  |  | Portugal | 25* |
| Normal HDL | 45.7 | 57.6 | 27.0 | 6.0 | n/a | 51 |  |  |
| Low HDL | 39.2 | 57.9 | 28.8 | 6.3 | n/a | 22 |  |  |
| Patients w/ risk factors for CVD |  |  |  |  |  |  |  |  |
| Normal HDL | 35.7 | 62.0 | 29.2 | 8.1 | n/a | 119 |  |  |
| Low HDL | 40.1 | 60.0 | 29.9 | 9.4 | n/a | 50 |  |  |
| Subjects w/o ESRD or CVD | 38.3 | 53.2 | 26.0 | n/a | none | 20 | Poland | 26 |
| ESRD on peritoneal dialysis | 27.3 | 52.8 | 25.5 | n/a | 25.0 | 52 |  |  |
| ESRD on hemodialysis | 30.8 | 57.7 | 25.0 | n/a | 14.8 | 54 |  |  |
| Type 2 diabetes |  |  |  |  |  |  | Spain | 27* |
| No atherosclerosis | 67.4 | 60.8 | 31.0 | 7.0 | n/a | 93 |  |  |
| Atherosclerosis | 70.4 | 64.9 | 29.0 | 7.0 | n/a | 73 |  |  |
| Patients w/o CHD event | 93.0 | 61.3 | 27.7 | n/a | none | 258 | Multi-national; men only | 14 |
| Patients w/ CHD event | 110.0 | 61.1 | 28.6 | n/a | none | 88 |  |  |

*Some subjects were taking lipid-lowering medications. oxLDL, oxidized LDL; BMI, body mass index; HbA1c, glycated hemoglobin; LDL, low-density lipoprotein; CAD, coronary artery disease; HDL, high-density lipoprotein; CVD, cardiovascular disease; ESRD, end-stage renal disease; CHD, coronary heart disease. All values are means or percent as indicated, reported by the corresponding study
